# RSFNet: A retention-based network with spatial–frequency joint enhancement for infrared small target detection

**DOI:** 10.1371/journal.pone.0345722

**Published:** 2026-08-06

**Authors:** Zhicheng Tan, Shanlin Sun, Guo Li

**Affiliations:** 1 School of Aeronautics and Astronautics, Guilin University of Aerospace Technology, Guilin, China; 2 Key Laboratory of Underwater Acoustic Communication and Marine Information Technology, Ministry of Education, School of Informatics, Xiamen University, Xiamen, Fujian, China; 3 Key Laboratory of Southeast Coast Marine Information Intelligent Perception and Application, Ministry of Natural Resources, Xiamen University, Xiamen, China; Institute of Wenzhou, Zhejiang University, CHINA

## Abstract

Infrared Small Target Detection (ISTD) is crucial for applications such as traffic monitoring and maritime surveillance. However, it remains highly challenging due to weak target signals and the absence of rich texture information, often resulting in low detection accuracy. Existing deep learning-based ISTD methods typically struggle to balance the trade-off between modeling long-range dependencies and avoiding feature oversmoothing. To this end, we propose RSFNet, a retention-based network with spatial–frequency joint enhancement for ISTD. RSFNet introduces a bidirectional 2D decay–retention attention mechanism into the vision Transformer (ViT) framework, which effectively suppresses background noise while capturing long-range dependencies. In addition, we design a Spatial–Frequency Joint Enhancement Module (SFE) to facilitate the transfer of salient target features from the encoder to the decoder. SFE integrates spatial and frequency domain features to facilitate global–local information interaction. Extensive experiments conducted on multiple publicly available ISTD datasets demonstrate that RSFNet significantly outperforms state-of-the-art (SOTA) methods in both detection accuracy and training efficiency, with a nearly 32% reduction in training time.

## 1. Introduction

Infrared Small Target Detection (ISTD) enables effective imaging under low-light conditions and has the capability to penetrate visual obstructions such as fog. It has attracted increasing attention across a range of critical applications, including traffic monitoring, early warning systems, and maritime surveillance [1–5]. Furthermore, by integrating underwater remote sensing technologies with infrared imaging, comprehensive ocean monitoring can be effectively realized, enabling enhanced perception and analysis of complex maritime environments [6–8]. Nevertheless, ISTD remains a highly challenging task. Due to long imaging distances, infrared targets typically appear extremely small—sometimes occupying only a single pixel—and inherently lack color and texture information. Furthermore, the significant attenuation of infrared radiation over distance leads to targets with very low contrast, which are often obscured by sensor noise and cluttered backgrounds, thereby hindering the reliable extraction of discriminative features.

Traditional ISTD methods can be broadly categorized into three types: filtering-based methods [9,10], local contrast-based methods [11–13], and low-rank-based methods [14–16]. These approaches typically begin by generating a confidence map that suppresses background clutter while highlighting potential targets. An adaptive thresholding technique is then applied to this map to extract the targets. However, due to the absence of feature learning capabilities, these methods often struggle to generalize to complex real-world scenarios.

With the rapid advancement of deep neural networks capable of automatically learning features from large-scale data encompassing complex scenes, CNN-based methods have demonstrated superior performance in ISTD compared to traditional approaches [17–19]. Liu et al. [20] proposed a generic detection framework by designing a five-layer multi-layer perceptron (MLP) network specifically for infrared small target detection. Wang et al. [21] decomposed the detection task into two sub-tasks, addressed by two adversarially trained models, achieving a balance between false alarms and missed detections. Dai et al. [22] introduced a novel method that integrates discriminative networks with conventional model-driven techniques, leveraging local contrast priors to enhance infrared small target detection. However, the inherent limitation of CNNs in capturing long-range dependencies remains a significant bottleneck, restricting further improvements in small target detection. To mitigate this issue, various studies have employed lateral or skip connections that link feature maps of different resolutions and semantic levels, as seen in architectures such as U-Net and SharpMask [23]. Among these approaches, Feature Pyramid Networks (FPNs) [24,25] have emerged as a prominent solution for enhancing long-range dependency learning in CNNs and have been widely adopted in segmentation tasks. In recent years, spectral neural networks have gained increasing attention. By leveraging the spectral convolution theorem from Fourier theory—which indicates that modifications in the spectral domain can globally influence all input features [26]—the integration of frequency and spatial domains has proven to be an effective strategy for extending the receptive field [27].

Building on Vision Transformers (ViTs) [28], another line of work is adept at modeling the long-range dependencies that CNNs struggle with, providing a more holistic understanding of complex scenes. Pan et al. [29] proposed an attention network with bilinear correlation, incorporating a convolution-linear fusion transformer to enhance target features and suppress noise. Yuan et al. [30] introduced spatial-channel transformer blocks and multi-scale feed-forward networks to encode semantic differences between targets and backgrounds, thereby enhancing the model’s hidden representations. Despite their strong performance, these transformer-based methods still face a significant challenge—target representation over-smoothing. Since infrared small targets occupy only a few pixels, their representations are prone to being blurred as network depth increases, due to the over-smoothing phenomenon [31,32]. This effect diminishes the model’s ability to distinguish targets from cluttered backgrounds, ultimately hindering detection performance.

The self-attention mechanism is a key defining characteristic of Transformer models. This mechanism can be viewed as a graph-like inductive bias that connects all tokens in a sequence through a relevance-based pooling operation [33]. Wang et al. [27] initially proposed non-local neural networks, which perform non-local operations by computing a position-specific response as a weighted sum of features at all positions. Chen et al. [34] proposed a new Deformable Patch to avoid destroying the semantics of objects. Wang et al. [35] proposed a cross-scale embedding layer and long-short distance attention, which together compensate for existing transformers’ inability to build cross-scale attention. Regarding computational efficiency, current approaches typically address the memory complexity challenges inherent in traditional attention mechanisms by utilizing sparse connection patterns [36], low-rank approximations [37], or recurrent operations [38]. In the field of infrared small target detection, Pan et al. [29] proposed an attention network with bilinear correlation, which includes a convolution-linear fusion transformer, to enhance target features and suppress noise. To suppress backgrounds and filter out false alarms, Wang et al. [39] proposed a coarse-to-fine interior attention-aware network (IAANet) for infrared small target detection. Yuan et al. [30] introduced spatial-channel transformer blocks and multi-scale feed-forward networks to encode semantic differences between targets and backgrounds, thereby enhancing the model’s hidden representations. Nevertheless, existing attention mechanisms are prone to diminishing the already scant target feature information. This challenge, caused by small pixel counts and dim features of small infrared targets, becomes more severe as network layers deepen. Another issue introduced by transformer-based approaches is their relatively slow convergence rate.

**Fig 1:**
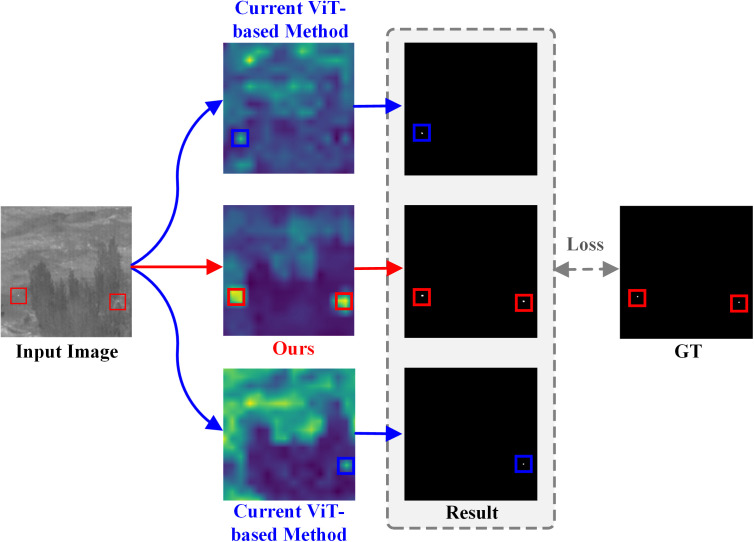
Compared with current ViT-based ISTD methods, RSFNet is able to learn the salient features of the target more accurately.

To address the above issues, we propose RSFNet, a retention-based network with spatial–frequency joint enhancement for the ISTD task. As shown in Fig 1, RSFNet demonstrates more robust detection performance compared to existing ViT-based ISTD methods. The main contributions of this work are summarized as follows:

- We propose a novel retention-based framework for infrared small target detection, termed RSFNet, which is specifically designed to enhance target representation under complex backgrounds and weak signal conditions.
- We introduce a novel 2D retention-based attention mechanism into the ViT architecture. By explicitly incorporating a target relevance mask, the proposed mechanism can effectively suppress background noise and alleviate the oversmoothing of salient target features, thereby enhancing the discriminative capability of target representations.
- We design a Spatial–Frequency Joint Enhancement Module (SFE) to improve feature interaction. SFE leverages the complementary advantages of spatial-domain local detail representation and frequency-domain global context modeling, enabling cross-domain interaction between global and local features and mitigating the loss of high-frequency information as the network deepens.
- Extensive experiments on three public ISTD datasets demonstrate the effectiveness of RSFNet.

The remainder of this paper is organized as follows. Section 2 describes the proposed method. Experimental results are presented in Section 3. Section 4 provides a detailed discussion, and conclusions are drawn in Section 5.

## 2. Methods

**Fig 2:**
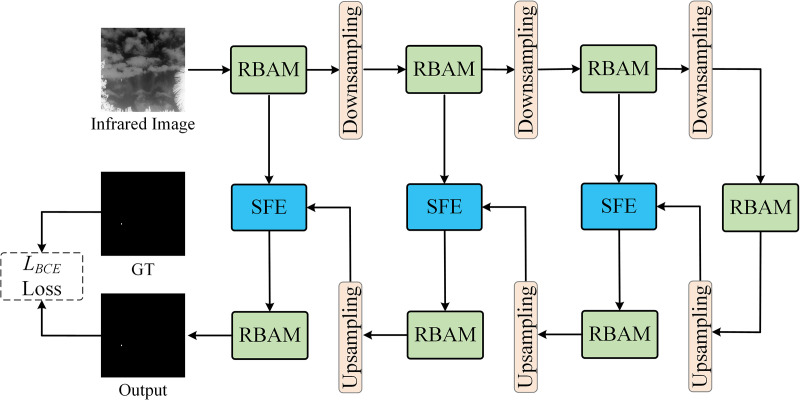
The overall architecture of RSFNet, which is a ViT-based network following the U-Net structure.

We propose a novel ISTD model named RSFNet, and its architectural overview is shown in Fig 2. In this section, we first briefly introduce the overall architecture in Section 2.1. Then, we present our newly designed Retention-Based Background Attenuation Module (RBAM) in Section 2.2, followed by the Spatial–Frequency Joint Enhancement Module (SFE) in Section 2.3. The details will be illustrated below.

### 2.1. Overall structure

As shown in Fig 2, RSFNet is a U-Net structured end-to-end network. It consists of three encoders, one bottleneck, and three decoders. The enhanced saliency information is transmitted between encoders and decoders at the same level through one SFE. For an input image of size H×W×C, it is first transformed into $\frac{h}{p}\times \frac{w}{p}$ patches with a channel dimension of *c* through a CNN-based patch embedding, where *p* denotes patch size.

### 2.2. Retention-based background attenuation module

The Retentive Network introduces an explicit decay mechanism into language models — a property that Transformers inherently lack. Retention models the sequence modeling problem in a recurrent manner, and can be formulated as:


$$\begin{array}{c} o_{n}=\sum_{m=1}^{n}\gamma^{n-m}\left(Q_{n}e^{in\theta }\right){\left(K_{m}e^{im\theta }\right)}^{†}v_{m}, \end{array}$$ (1)

when γ is a constant, Eq. (1) is rewritten as:


$$\begin{array}{c} Q=\left(XW_{Q}\right)⊙\Theta ,\;K=\left(XW_{K}\right)⊙\bar{\Theta },\;V=XW_{V},\;\Theta_{n}=e^{in\theta },\;D_{nm}=\left\{ \begin{array}{cc} \gamma^{n-m}, & n\geq m\\ 0, & n<m \end{array}\right.\;Ret(X)=\left(QK^{⊤}⊙D\right)V, \end{array}$$ (2)

where $\bar{\Theta }$ is the complex conjugate of Θ, and $D\in {\mathbb{R}}^{\left|x|\times |x\right|}$ combines causal masking and exponential decay along relative distance as one matrix, which brings additional a prior knowledge. For ISTD, we first extend the unidirectional, explicit decay self-attention mechanism from RetNet to a bidirectional, 2D form, Enables more target-relevant feature information to be accurately captured and utilized. The new 2D form attention is defined as:


$$\begin{array}{c} o_{n}=\sum_{m=1}^{N}\gamma^{\left|n-m\right|}\left(Q_{n}e^{in\theta }\right){\left(K_{m}e^{im\theta }\right)}^{†}v_{m}, \end{array}$$ (3)

where *N* is the number of tokens. The equation can be rearranged into a parallel form, expressed as:


$$\begin{array}{c} BiRet(X)=\left(QK^{⊤}⊙D^{Bi}\right)⊙V,\;D^{Bi}=\gamma^{\left|n-m\right|},\; \end{array}$$ (4)

where BiRet(·) denotes the retention with bidirectional modeling ability. For images, each token has a unique 2D coordinate on the plane. For the *n*-th token, we use $\left(x_{n},y_{n}\right)$ to represent its 2D coordinate. Based on the 2D coordinates of all tokens, we modify each element in matrix *D* to be the Manhattan distance between the corresponding token pairs at their respective positions, thereby completing the transformation from a 1D to a 2D decay coefficient. We then obtain the 2D Retentive Attention as follows:


$$\begin{array}{c} Ret^{2D}(X)=\left(QK^{⊤}⊙D^{Bi}\right)V,\;D^{Bi}=\gamma^{\left|x_{n}-x_{m}\right|+\left|y_{n}-y_{m}\right|},\; \end{array}$$ (5)

**Fig 3:**
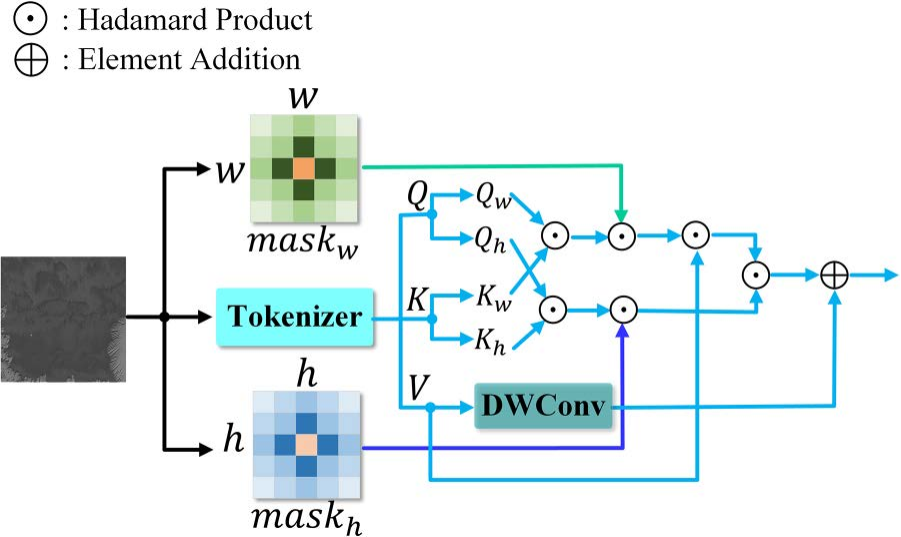
The detailed designs of RBAM.

The detailed designs of RBAM is shown in Fig 3. A challenging issue in Transformer design is that global self-attention is computationally expensive, whereas local self-attention often limits the interaction range of each token.

**Fig 4:**
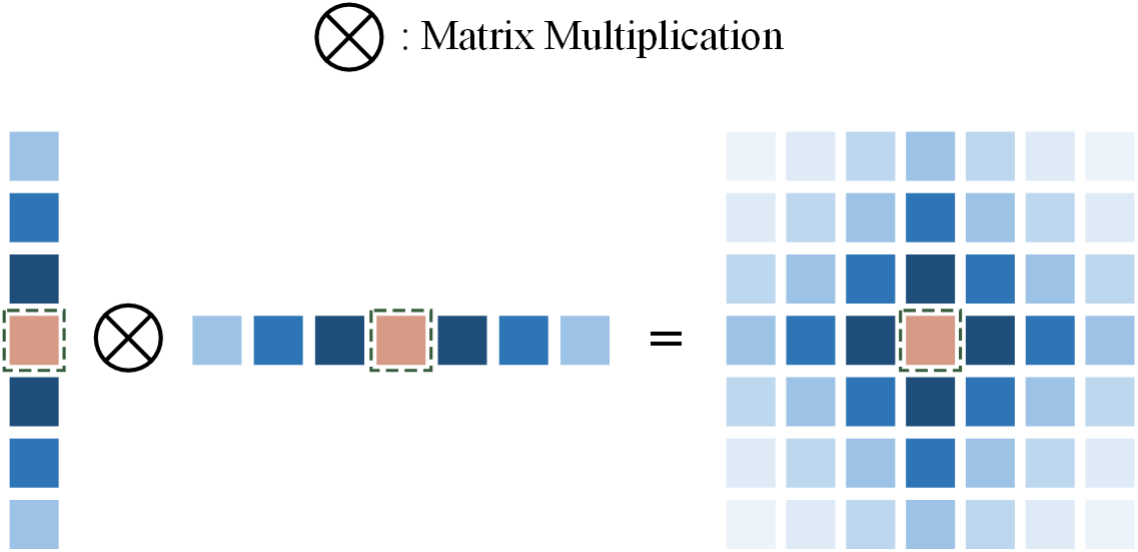
Perform self-attention in horizontal and vertical stripes simultaneously.

To address this issue, CSWin [40] develops the Cross-Shaped Window self-attention mechanism, which computes self-attention in horizontal and vertical stripes in parallel, and achieves strong modeling capability while limiting computational cost. Inspired by the design of CSWin Self-Attention, we propose efficient self-attention mechanisms that explicitly model local features based on a two-dimensional coordinate system. As shown in Fig 4, The input feature $X\in R^{\left|H|\times |W\right|}$ is first linearly projected into *K* heads, after which each head performs self-attention within either horizontal or vertical stripes. The bidirectional, two-dimensional explicit decay *M*^2*d*^ is incorporated into self-attention:


$$\begin{array}{c} ℛ(X)=\left(Q_{w}K_{w}^{⊤}⊙M^{2d}\right)⊙\left(Q_{h}K_{h}^{⊤}⊙M^{2d}\right)V,\;M^{2d}=\gamma^{\left|x_{n}-x_{m}\right|+\left|y_{n}-y_{m}\right|},\; \end{array}$$ (6)

$M^{2d}\in {\mathbb{R}}^{\left|x|\times |x\right|}$ is a 2D decay coefficient that contains relative position information while discarding causal information compared to $D\in {\mathbb{R}}^{\left|x|\times |x\right|}$ in RetNet. Specifically, based on the 2D coordinates of each token, we modify each element of matrix *D* to be the Manhattan distance between the corresponding token pairs at their respective positions. The specific process is as follows:


$$\begin{array}{c} Q_{h},Q_{w}=Split(Q),K_{h},K_{w}=Split(K),\;\hat{Q}=Q_{w}K_{w}^{⊤},\hat{K}=Q_{h}K_{h}^{⊤},\;X=\left(\hat{Q}⊙{M_{h}}^{2d}⊙\hat{K}⊙{M_{w}}^{2d}\right)V,\;X_{out}=Linear(Cat(X_{i})), \end{array}$$ (7)

We adopt the method used by [40] to split *Q* and *K*, and then concatenate the $X_{i}$ obtained after computing the multi-head self-attention.

RBAM helps the network focus more on target-related regions while suppressing irrelevant background interference. By introducing a 2D distance-aware decay into stripe attention, it preserves useful long-range dependencies while preventing weak small-target features from being overwhelmed by global background responses. Its workflow can be summarized as follows: Input feature map → generate Q, K, V → split attention into horizontal and vertical stripes → apply 2D distance-decay weighting → fuse attended features → output refined feature map.

### 2.3. Spatial–frequency joint enhancement module

Although RBAM effectively reduces the network’s sensitivity to background interference and enhances its focus on small targets, the increasing network depth still introduces a degree of information loss, which in turn limits the overall performance of target detection. To address this, we design the Spatial–Frequency Joint Enhancement Module (SFE), aiming to enable cross-domain interaction between global and local features by leveraging the complementary strengths of spatial-domain local detail representation and frequency-domain global context modeling.

The spectral convolution theorem in Fourier theory reveals that modifying a single point in the frequency domain affects all input features globally. Building on this insight, we propose a spatial–frequency joint enhancement module based on Fourier convolution, which leverages an image-level receptive field and integrates global and local receptive fields through dual-domain interaction between the spatial and frequency domains. This interaction not only enhances the feature representation of dim targets but also significantly improves the contrast between targets and their surrounding background.

For the input feature map *f*, it is first split into global features $f_{g}$ and local features $f_{l}$:


$$\begin{array}{c} f_{g},f_{l}=split(f), \end{array}$$ (8)

**Fig 5:**
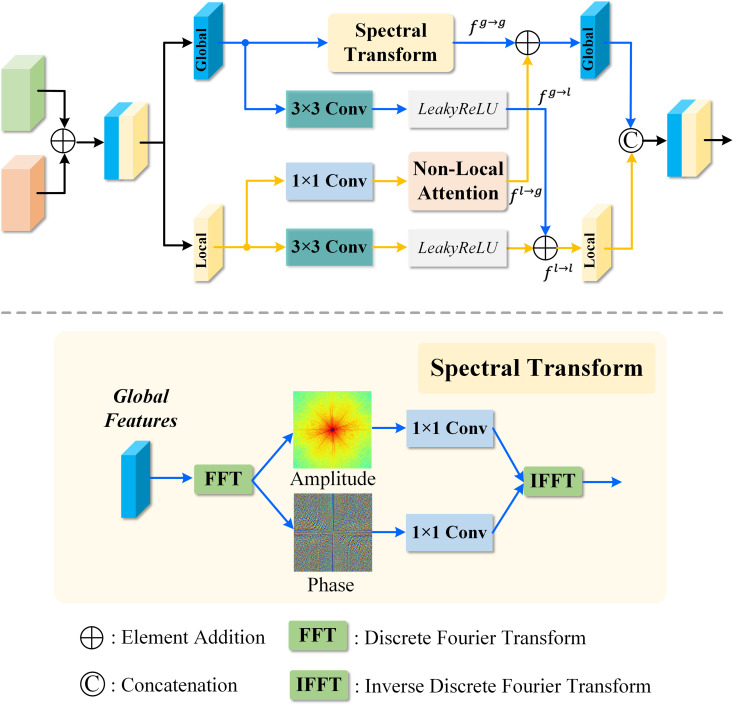
The detailed designs of SFE.

As shown in Fig 5, the module comprises four branches: local-to-local, local-to-global, global-to-local, and global-to-global—which enable mixed receptive fields via interaction across the spatial-frequency dual domain. Both the local-to-local ($f^{l\to l}$) and global-to-local ($f^{g\to l}$) branches capture local features using a 3×3 convolution, The local-to-global branch ($f^{l\to g}$) employs a non-local attention mechanism to explore global dependencies for each query pixel in relation to its surrounding regions, while the global-to-global branch ($f^{g\to g}$) utilizes the Fourier transform to expand the receptive field and capture long-range context. The procedures can be described as follows:


$$\begin{array}{c} f^{g\to g}\left(f_{g}\right)=ST\left(f_{g}\right),\;f^{g\to l}\left(f_{g}\right)={\mathrm{Conv}}_{3\times 3}^{g\to l}\left(f_{g}\right),\;f^{l\to g}\left(f_{l}\right)=NL\left({\mathrm{Conv}}_{1\times 1}^{l\to g}\left(f_{l}\right)\right),\;f^{l\to l}\left(f_{l}\right)={\mathrm{Conv}}_{3\times 3}^{l\to l}\left(f_{l}\right), \end{array}$$ (9)

where NL denotes the non-local attention mechanism, and ST is the spectral transformation which utilizes the Fourier transform to efficiently expand the receptive field to cover the full resolution of the input feature map. We first apply the 2D Fast Fourier Transform (FFT) to transform spatial features into the frequency domain, producing both real and imaginary components. Subsequently, a 3×3 convolution followed by a LeakyReLU activation — denoted as operation 𝒪 is applied before transforming the features back to the spatial domain via the inverse Fourier transform:


$$\begin{array}{c} R\left(f_{g}\right),I\left(f_{g}\right)=ℱ\left(f_{g}\right),\;R\left(f_{g}\right)=𝒪\left(R\left(f_{g}\right)\right),\;I\left(f_{g}\right)=𝒪\left(I\left(f_{g}\right)\right),\;f_{st}={ℱ}^{-1}(R(f_{g}),I(f_{g})), \end{array}$$ (10)

where ℱ and ${ℱ}^{-1}$ denote the Fourier transform and inverse Fourier transform, respectively. *R* and *I* represent the real and imaginary components of the signal. By summing the features from the global-to-global and local-to-global branches, we obtain the global feature, and in the same way, we obtain the local features from the other two branches.


$$\begin{array}{c} F_{g}=f^{g\to g}\left(f_{g}\right)+f^{l\to g}\left(f_{l}\right)=f_{st}+f^{l\to g}\left(f_{l}\right),\;F_{l}=f^{g\to l}\left(f_{g}\right)+f^{l\to l}\left(f_{l}\right),\; \end{array}$$ (11)

where $F_{g}$ and $F_{l}$ denote the global and local features, respectively. These features are then concatenated to generate the enhanced representation *F*.


$$\begin{array}{c} F=Cat\left(F_{g},F_{l}\right). \end{array}$$ (12)

SFE is used to strengthen target information that may be weakened in deep feature extraction. It combines spatial-domain local details with frequency-domain global context, so that dim small targets can be represented more clearly and transferred more effectively from encoder to decoder. Its workflow can be summarized as follows: Input feature map → split into local and global branches → extract local details in the spatial domain → capture global context in the frequency domain → perform cross-branch enhancement → concatenate enhanced features → output strengthened feature map.

### 2.4. Loss function

Dice loss [41] measures the similarity between the predicted image and the ground truth:


$$\begin{array}{c} {ℒ}_{dice}=1-\frac{2\cdot \sum_{i=1}^{N}y_{i}{\hat{y}}_{i}}{\sum_{i=1}^{N}y_{i}+\sum_{i=1}^{N}{\hat{y}}_{i}}. \end{array}$$ (13)

where ${\hat{y}}_{i}$ represents the predicted mask, and $y_{i}$ represents the corresponding ground truth mask.

## 3. Results

### 3.1. Baseline methods

To evaluate the effectiveness of our method, we compared it with eleven deep learning-based ISTD methods, including ALCNet [22], ISNet [42], DNA-Net [43], AGPCNet [44], RDIAN [45], IAANet [39], ABC [29], SCTransNet [30], PBTNet [46], HDNet [47], and SFDTNet [48].

### 3.2. Datasets and implementation details

#### 3.2.1. Implementation details.

We implemented our network on a PC equipped with a single NVIDIA GeForce RTX 3090 GPU, using the PyTorch framework with CUDA 11.6 and PyTorch 1.12. The learning rate is initially set to $1\times 10^{-4}$ and gradually reduced to $1\times 10^{-6}$ in a linear decay schedule as training progresses. The batch size was set to 4. The model is trained for 200 epochs using the Adam optimizer. All baseline methods followed the settings reported in their original papers, without any additional method-specific adjustments, so as to ensure the fairness and comparability of the experimental results.

#### 3.2.2. Datasets.

To evaluate the performance of our model, we conducted experiments on two real infrared small target detection datasets: NUAA-SIRST [49] and IRSTD-1K [42]. To enrich environmental contexts and obtain more precise pixel-level annotations than manual labeling, we also performed experiments on a synthetic dataset named NUDT-SIRST [43]. Specifically, the NUAA-SIRST dataset contained 427 images and 480 instances drawn from hundreds of real-world scenes captured with different infrared sequences. The IRSTD-1K dataset consisted of 1,000 diverse images, primarily focusing on target shape annotations, thus providing abundant supervisory signals. Additionally, the NUDT-SIRST dataset comprised 1,327 images generated by synthesizing real infrared backgrounds with various simulated infrared targets. In this paper, we adopt a random split strategy to divide the dataset into training and test sets. Specifically, the test set is randomly sampled, and all remaining samples are used for training. Following [22], the train-to-test ratio was set to 3. The random seed is set to 42. All experiments are repeated three times, and the final results are reported as the average of the three runs.

#### 3.2.3. Evaluation metrics.

To provide a comprehensive and fair performance comparison between SOTA methods and our model, we employ intersection over union (*IoU*) and normalized IoU (*nIoU*) to evaluate shape description capacity, alongside the probability of detection $\left(P_{d}\right)$ and the false alarm rate $\left(F_{a}\right)$ to evaluate localization performance. Concretely, *IoU* measures the overlap between predicted targets and true targets, thereby evaluating the shape description capability of the algorithms.


$$\begin{array}{c} IoU=\frac{\sum_{i=1}^{N}TP_{i}}{\sum_{i=1}^{N}\left(T_{i}+FP_{i}\right)}, \end{array}$$ (14)

where *TP* denotes the number of target pixels correctly detected as true positives, *FP* indicates the number of background pixels mistakenly predicted as target pixels, *T* represents the number of true target pixels, and *N* refers to the total number of pixels. Similarly, *nIoU*, as a supplementary metric, provides a better balance between pixel accuracy and structural similarity of the infrared target.


$$\begin{array}{c} nIoU=\frac{1}{N}\sum_{i=1}^{N}\frac{TP_{i}}{T_{i}+FP_{i}}, \end{array}$$ (15)

Furthermore, $P_{d}$ is extensively employed to assess detection effectiveness for small and dim infrared targets, quantifying the ratio of correctly detected targets to the total number of targets.


$$\begin{array}{c} P_{d}=\frac{TP}{TP+FN}, \end{array}$$ (16)

$F_{a}$ is utilized to evaluate false detection by measuring the ratio of falsely predicted pixels to all pixels $N_{all}$,


$$\begin{array}{c} F_{a}=\frac{FP}{N_{all}}. \end{array}$$ (17)

where *FN* is the number of false negative pixels.

### 3.3. Quantitative comparison

Table 1–3 present the performance of our proposed method compared with baseline methods on three datasets. Our method outperformed existing ISTD approaches across all evaluation metrics. Specifically, compared with the second-best results, our method improves nIoU by 0.35, 0.89, and 0.05 percentage points on the NUAA-SIRST, IRSTD-1K, and NUDT-SIRST datasets, respectively. Table 4 presents the comparison of inference time with SOTA methods. It can be observed that RSFNet achieves an inference speed of 39.33 ms per frame at a resolution of 256×256 while maintaining the best performance, approaching real-time processing at 24 FPS.

**Table 1:** Performance Comparison on the NUAA-SIRST Dataset.

Method	IoU (↑)	nIoU (↑)	$P_{d}$ (↑)	$F_{a}$ (↓)
	(%)	(%)	(%)	($\times 10^{-6}$)
ALCNet	66.93	67.25	92.66	10.56
ISNet	71.71	73.18	99.08	39.06
DNA-Net	74.89	74.51	98.17	8.81
AGPCNet	70.60	70.16	97.25	37.44
RDIAN	69.86	70.61	96.33	26.34
IAANet	64.13	67.25	91.74	9.53
ABC	68.12	69.06	93.58	16.58
SCTransNet	73.05	74.13	95.37	11.84
PBTNet	73.47	74.33	97.11	11.90
HDNet	74.01	74.83	98.62	10.93
SFDTNet	74.77	75.39	98.63	10.22
**Ours**	**75.56**	**75.74**	**99.39**	**8.15**

**Table 2:** Performance Comparison on the IRSTD-1K Dataset.

Method	IoU (↑)	nIoU (↑)	$P_{d}$ (↑)	$F_{a}$ (↓)
	(%)	(%)	(%)	($\times 10^{-6}$)
ALCNet	62.63	60.70	89.23	19.28
ISNet	61.80	62.27	89.56	26.25
DNA-Net	62.66	60.86	90.24	9.56
AGPCNet	62.82	63.01	90.57	29.72
RDIAN	58.33	60.73	91.92	26.53
IAANet	60.28	57.00	89.90	18.11
ABC	62.49	61.91	91.88	21.65
SCTransNet	61.94	61.71	89.22	30.71
PBTNet	62.77	62.55	91.10	21.88
HDNet	63.57	63.30	91.28	18.09
SFDTNet	64.80	64.91	92.73	16.19
**Ours**	**65.91**	**65.80**	**92.26**	**7.32**

**Table 3:** Performance Comparison on the NUDT-SIRST Dataset.

Method	IoU (↑)	nIoU (↑)	$P_{d}$ (↑)	$F_{a}$ (↓)
	(%)	(%)	(%)	($\times 10^{-6}$)
ALCNet	76.35	77.53	96.96	16.78
ISNet	74.72	74.69	93.91	21.64
DNA-Net	84.00	84.23	97.17	4.09
AGPCNet	85.48	86.60	98.04	7.12
RDIAN	73.04	74.59	95.65	22.11
IAANet	73.33	75.86	93.26	52.88
ABC	84.65	84.33	97.83	21.51
SCTransNet	92.15	89.35	98.43	3.62
PBTNet	92.61	91.76	97.60	18.46
HDNet	89.08	91.99	98.30	7.29
SFDTNet	93.40	92.48	98.22	16.33
**Ours**	**93.44**	**92.53**	**98.69**	**3.04**

**Table 4:** Comparison of inference time with SOTA methods.

Method	ALCNet	DNA-Net	RDIAN	ABC	SCTrans	PBTNet	HDNet	SFDTNet	Ours
Infer. (ms)	7.78	32.44	6.06	72.49	37.97	140.61	17.65	33.64	39.33

### 3.4. Visual comparison

**Fig 6:**
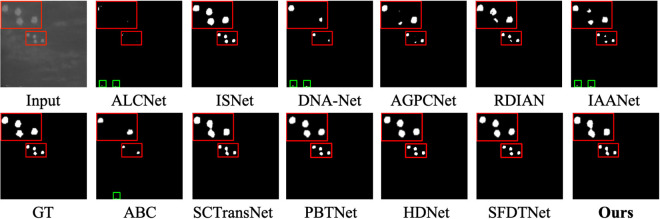
Visual comparison on the NUAA-SIRST dataset.

**Fig 7:**
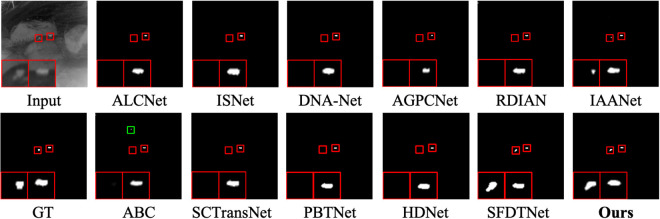
Visual comparison on the IRSTD-1K dataset.

**Fig 8:**
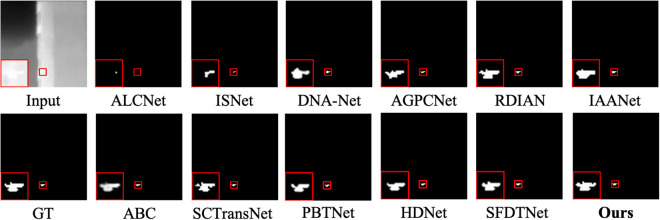
Visual comparison on the NUDT-SIRST dataset.

We present the detection results of representative samples from the NUAA-SIRST, IRSTD-1K, and NUDT-SIRST datasets, as illustrated in Figs 6, 7, and 8, respectively. Our RSFNet achieved more accurate target boundary delineation and exhibited better robustness across different datasets.

### 3.5. Ablation experiments

To verify the effectiveness of our proposed key modules, we conducted ablation experiments on the NUDT-SIRST dataset. The experiments focused on two key modules, RBAM and SFE. We first performed an overall ablation of the two modules, and then individually removed their core components to verify their effectiveness. Table 5–7 present the results of the ablation experiments.

**Table 5:** Ablation Study of RBAM and SFE Modules on the NUDT-SIRST Dataset.

Config.	RBAM	SFE	IoU (↑)	nIoU (↑)	$P_{d}$ (↑)	$F_{a}$ (↓)
			(%)	(%)	(%)	($\times 10^{-6}$)
(II) RBAM Only	✓	✗	84.71	84.16	93.91	118.10
(III) SFE Only	✗	✓	80.77	79.16	97.82	102.70
(IV) **Full Model**	✓	✓	**93.44**	**92.53**	**98.69**	**3.04**

*Note:* ✓ = module included, ✗ = module excluded. RBAM = Retention-Based Attention Module; SFE = Spatial–Frequency Enhancement. IoU = Intersection over Union; $P_{d}$ = Detection Probability; $F_{a}$ = False Alarm Rate.

**Table 6:** Ablation Study of the 2D Decay–Retention Attention Mechanism on the NUDT-SIRST Dataset.

Config.	IoU (↑)	nIoU (↑)	$P_{d}$ (↑)	$F_{a}$ (↓)
	(%)	(%)	(%)	($\times 10^{-6}$)
Without Attention (✗)	79.90	79.88	68.91	31.02
With Attention (✓)	**93.44**	**92.53**	**98.69**	**3.04**

*Note:* ✓ = module included, ✗ = module excluded. Higher is better for IoU, nIoU, and $P_{d}$; lower is better for $F_{a}$.

**Table 7:** Ablation Study of Spectral Transform (ST) and Non-Local Attention (NLA) on the NUDT-SIRST Dataset.

Config.	ST	NLA	IoU (↑)	nIoU (↑)	$P_{d}$ (↑)	$F_{a}$ (↓)	FLOPs	Mem
			(%)	(%)	(%)	($\times 10^{-6}$)	(G)	(GB)
(I) Only NLA	✗	✓	79.34	78.12	97.69	30.88	17.91	0.65
(II) Only ST	✓	✗	80.13	80.23	95.43	39.75	**17.30**	**0.60**
(III) **Ours (ST + NLA)**	✓	✓	**93.44**	**92.53**	**98.69**	**3.04**	18.44	0.68

*Note:* ✓ = module included, ✗ = module excluded. ST = Spectral Transform, NLA = Non-Local Attention. Higher is better for IoU, nIoU, and $P_{d}$; lower is better for $F_{a}$.

## 4. Discussion

### 4.1. Comparison with SOTA methods

In Fig 6, and 7, the complex scenes generated numerous pseudo-targets that closely resembled real ones, leading to prominent false alarms and missed detections by other methods. In contrast, our approach demonstrated superior robustness and precision in handling such challenging scenarios. Fig 8 illustrates a case where the contrast between the target and background is extremely low, posing a significant challenge to existing methods in accurately capturing fine details. While many prior approaches successfully located the targets, their representations of target shapes remained coarse. In comparison, our method achieved a substantial improvement in segmentation quality.

**Fig 9:**
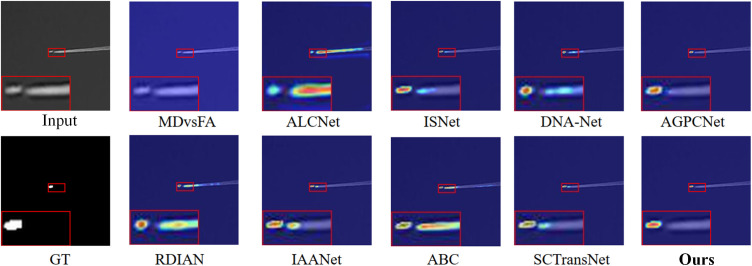
Visual comparison of heat map after 50 epochs of training on the IRSTD-1K dataset.

**Fig 10:**
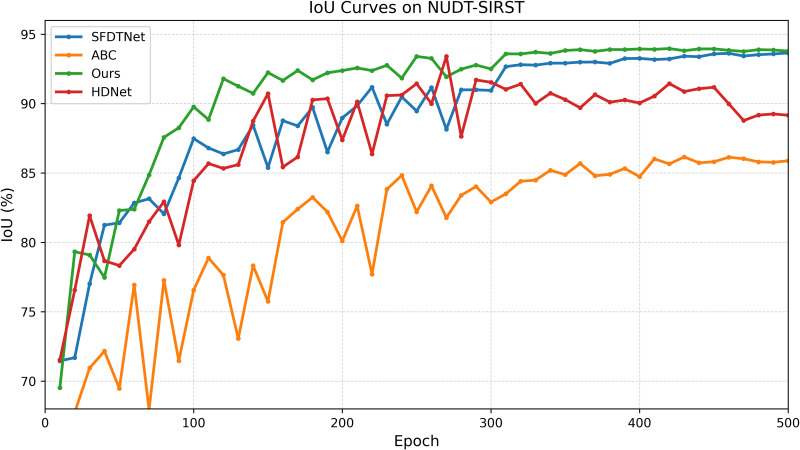
IoU convergence curves on the NUDT-SIRST dataset, where the x-axis represents the training epoch and the y-axis represents the IoU score.

In Fig 9, we trained ten deep learning-based ISTD methods under identical conditions for 50 epochs and visualized the corresponding attention heatmaps. The results revealed that many methods failed to focus attention on the target, with some even struggling to capture its fundamental features. In contrast, our RSFNet rapidly concentrated attention around the target, effectively preserving critical feature information and improving training efficiency. This performance gain was attributed to our proposed target-oriented attention mechanism based on two-dimensional explicit decay, which efficiently suppressed irrelevant attention and enhanced overall model performance. Fig 10 presents the IoU curves on the NUDT-SIRST dataset, showing that our proposed RSFNet converges faster than the other methods.

### 4.2. Ablation experiments

To assess the impact of the two key modules, we followed a stepwise evaluation approach. Table 5 illustrates the relative importance of each module. When RBAM was removed, the performance decreased by 13.5%, 14.4%, and 0.8% in terms of IoU, nIoU, and $P_{d}$, respectively. When SFE was removed, the performance decreased by 9.3%, 9.0%, and 4.8%, respectively.

RBAM aims to address the challenge of vanishing small target features as network depth increases, and to efficiently process voluminous irrelevant background information in the ISTD task, thereby refining the Transformer’s attention layer for more precise targeting. The key component of RBAM is the 2D explicit decay mechanism, whose effectiveness is evaluated by removing it. As shown in Table 6, the 2D explicit decay mechanism significantly boosts the performance of RBAM.

To better understand SFE, we performed ablation experiments on its two key components: spectral transform (ST) and non-local attention (NLA). For SPT, we replaced it with a 3×3 convolution to evaluate its contribution. For NLA, we removed it in this variant and directly concatenated features for subsequent processing. As shown in Table 7, NLA and ST account for approximately 6.18% and 2.87% of the additional computational cost, while yielding significant nIoU improvements of about 15.57% and 13.29%, indicating that both components contribute positively to the construction of SFE.

## 5. Conclusion

Infrared small target detection (ISTD) plays a crucial role in various applications such as traffic monitoring, early warning systems, and maritime surveillance. However, ISTD faces several challenges. On one hand, small targets captured by infrared sensors are often located at extremely long distances, and the infrared radiation signals decay significantly with distance, resulting in only faint target signals being obtained. On the other hand, due to the susceptibility to background noise, infrared small targets typically exhibit low contrast, lacking discriminative information such as texture and color. These factors collectively make the detection of infrared small targets particularly difficult.

Existing deep learning-based ISTD methods can be broadly categorized into CNN-based and ViT-based approaches. CNN-based methods are effective at capturing local correlations but have limited capability in modeling long-range dependencies. ViT-based methods compensate for this limitation by capturing global context, but their computational complexity grows quadratically, and deeper networks tend to cause over-smoothing of features, which restricts further improvement in detection accuracy.

To address the aforementioned challenges, we propose RSFNet, a retention-based network with spatial–frequency joint enhancement for ISTD. RSFNet is designed as a U-Net-style architecture to ensure robust feature representation. Built upon the ViT framework, RSFNet incorporates a novel 2D retention-based attention mechanism that guides the attention weights to concentrate on the target regions while gradually decaying toward the surrounding areas. This mechanism helps suppress background noise and enhances the target’s discriminability. Furthermore, to mitigate the loss of high-frequency information caused by increased network depth, we introduce a Spatial–Frequency Joint Enhancement Module (SFE). SFE enables cross-domain interaction between global and local features by leveraging the complementary strengths of spatial-domain local detail representation and frequency-domain global context modeling. The features from the encoder are enhanced by SFE before being passed to the decoder, thereby compensating for the loss of discriminative information crucial for detecting small targets.

We conducted extensive experiments on the NUAA-SIRST, IRSTD-1K, and NUDT-SIRST datasets. The experimental results demonstrate that RSFNet outperforms state-of-the-art methods (SOTA) in detection performance and exhibits faster convergence.

In future work, we will develop a lightweight version of RSFNet to reduce computational cost and facilitate its real-time deployment on resource-constrained platforms.
